# Spatio-temporal diffusion of residential land prices across Taipei regions

**DOI:** 10.1186/2193-1801-3-505

**Published:** 2014-09-08

**Authors:** Anupam Nanda, Jia-Huey Yeh

**Affiliations:** Henley Business School, University of Reading, Reading, UK

**Keywords:** Residential land price, Ripple effect, Panel data, Spatial autocorrelation

## Abstract

**Abstract:**

Past studies have shown that changes in the house price of a region may transmit to its neighbouring regions. The transmission mechanism may follow spatial and temporal diffusion processes. This paper investigates such regional housing market dynamics and interactions among local housing sub-markets in Taipei. The analysis is based on a panel data framework and spatial panel models using annual data on median residential land prices from 41 Taipei sub-markets over the period from 1992 to 2010. The empirical analysis suggests that spatial dependence plays a significant role in interactions among regional housing markets. The results are strongly robust across several model specifications and regions controlling for time fixed effects and space-time covariance. These findings have significant implications for urban spatial planning and efficient use of public resources in mega-urban areas.

**JEL classifications:**

C21; C23; R12; H50

## 1 Introduction

House prices vary over space and time. Due to growing integration across regional economies, house price shocks to the central area are likely to propagate to the surrounding areas and then reach the peripheral regions. This is termed as the ‘ripple’ effect in the literature. A large volume of literature has focused on the ripple effect or diffusion of house prices and many studies have revealed significant lead-lag relations among movements of house prices in neighbouring regions. The efficient market hypothesis (EMH) implies that two equivalent assets should have the same market prices. However, housing markets are far from being fully efficient due to high transaction costs, infrequent transactions, lumpy investments and a high degree of heterogeneity in sub-market characteristics. Therefore, there may exist significant arbitrage opportunities across regional housing markets. As Pollakowski and Ray (1997) suggest, there is a ‘positive feedback effect’ implying that the house price in one region is not only determined by its own lagged prices but also by the adjacent regions’ lagged house prices.

This paper aims to investigate the dynamics of inter-urban house prices, especially to what extent the housing sub-markets converge or diverge over time. This paper uses a panel data framework and dynamic spatial panel data models, which combines spatial autocorrelation with panel data to estimate correlations of housing prices among different areas over time. We test several competing specifications and samples.

This paper makes three main contributions as follows. First, although several papers have examined the house price diffusion process in developed country set-up, the evidence from Asia is rather scant. The Asian experience may be quite different and interesting given the rapid urbanization and prevalence of mega-urban regions. This paper offers a rigorous analysis of a major Asian market (Taipei). Second, although previous studies have dealt with varying levels of methodological complexities and rigour, several sources of estimation biases still remain and those can significantly undermine much of the findings. In this paper, we employ latest methodological innovation in space-time panel models to study diffusion process of the residential land prices in Taipei. The application of this method brings out several interesting relationships while addressing several serious estimation issues, which may not have been revealed through the use of standard techniques. Third, the results not only reveal interesting spatial pattern of house prices, but also show causal relationships across specific local housing markets. The findings show importance of centralised economic development in influencing residential land values.

An explanation for inter-linkages of regional house prices may be put forward by focusing on the neighbourhood effects. House prices are not only affected by the characteristics of the property but also by the surrounding neighbourhood attributes. Positive changes in characteristics in nearby structures would create a positive feedback effect on the market values of the houses, thereby creating a *spatial dependence* (Can 1990). Ioannides and Zabel (2003) point out that the impact of neighbourhood effects can lead to changes in housing demands because these factors may affect consumers’ housing location choices.

Moreover, the possibility of substitution across areas might lead to lead-lag movements in house prices. In market-oriented economies, land values and land uses can be bid by potential users, and the property rights can be assigned accordingly. These characteristics would be an incentive to redevelop land leading to changes in highest and best land use. Therefore, land values in central locations represent willingness to pay and are determined by saving in travelling cost (Bertaud and Malpezzi 2003). However, higher house prices in central areas might cause urban expansion or urban sprawl, which in turn, could raise house prices in suburban and peripheral areas. High house prices in the central regions lead to higher demand for cheaper houses or larger residential space in suburbs and peripheral areas. This increase in the demand for suburban housing could push up the house prices over time.

**Figure 1:**
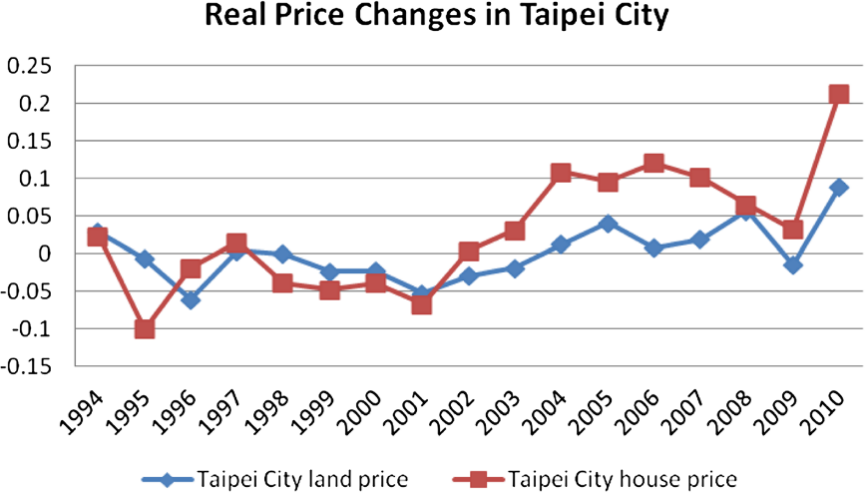
**Real residential land price changes in Taipei City.**

**Figure 2:**
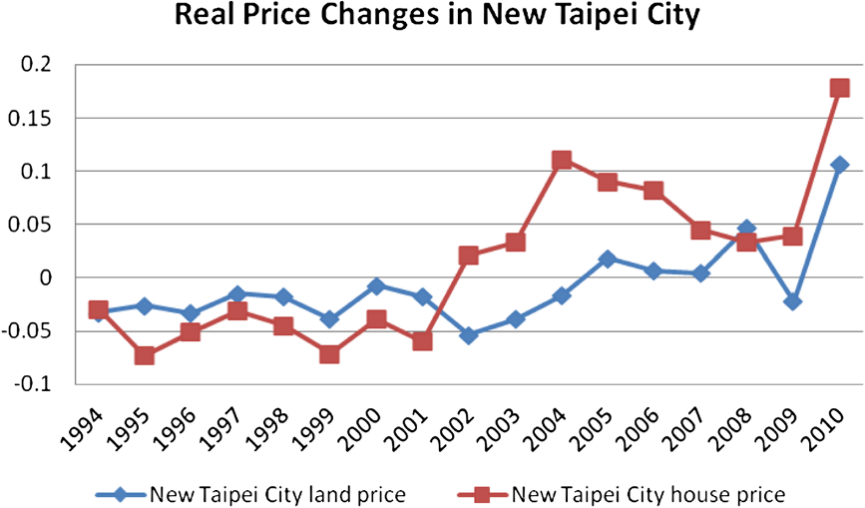
**Real residential land price changes in New Taipei City.**

While the local market demand-supply interactions and imbalances may primarily determine house price movements (Canarella et al. 2012), the interregional transmission of shocks in house prices also plays an important role in co-movement of house prices (Meen 1999). There are also considerable empirical evidences showing spatial dependence of regional house prices across a number of countries. In simple terms, such inter-relations among housing markets within a close proximity are driven by a multitude of economic and demographic factors e.g. local economic development, infrastructure improvement, migration patterns, cost of housing, urban form, active government policies and urban spatial planning. A clear understanding of these diffusion processes are important for purposes of policy making at the regional and local government levels and also, for a more efficient use of public resources. In this paper, our aim is to understand the diffusion processes of residential land prices in Taipei, a major metropolitan area in Taiwan.The housing markets in Taiwan may potentially reveal lead-lag movements between Taipei and other regions. Taipei, the dominant regional economy of Taiwan, attracts industries and boosts inter-regional migration from other parts of the country. This phenomenon increases the demand for housing and thus pushes up the house prices. Since 2003, the house prices in Taipei City have experienced significant increases of almost 161% in nominal terms, and in the surrounding region of New Taipei City, an increase of 136% over the same period of 2003 to 2011 has been observed. In 2011, the population in Taipei region accounted for around 29% of the total population in the country, and the per capita disposable income was over 1.4 times the national average. However, due to the high level of house prices and restrictive land regulations in the central areas, more people are forced to move into suburbs or peripheral areas. This may have contributed to urban sprawl and substantial increases in house prices in these areas. Moreover, Taiwan’s planning system is divided into urban planned districts and non-urban planned districts for land use control. Within Taipei city (urban planned districts), several strict and inflexible zoning rules were introduced in 1984. In order to restrict the expansion in the non-urban districts, a land use control measure was introduced in 1990. However, the legislation was not implemented until 1997 due to the pressure from the public interest groups, apprehending a negative impact on housing developments. Consequently, during 1990 to 1997, new construction in the Taipei city’s peri-urban areas increased dramatically possibly due to the expectation of high land costs. However, the subsequent oversupply also led to almost a ten-year long stagnant land prices (see Figure 1 and Figure 2).

Since Taipei plays a dominant role in the national economy, it is worth investigating movements in Taipei’s housing market and whether the ripple effects across Taipei regions exist. Moreover, housing markets cannot be seen as being aggregated into one national or regional market because of various circumstances. For instance, characteristics of different land uses could generate different movements in local markets. Taipei area, being highly dense and having restrictive zoning regulations, makes an interesting case study to examine land price diffusion process. Moreover, the Asian urbanization process appears to be unique in its dynamics and characteristics. There is now a growing literature around how the Asian urbanization process differs from standard developed country experiences. As McGee (1991) aptly pointed out that the Western paradigm of the urban transition is not applicable to the developing countries’ urbanization process and it has resulted in divergent patterns of urbanization. The Asian urbanization process is uniquely characterised by dominance of farm sector, rapid depopulation of rural areas, labour cost arbitrage, rural-urban income inequality, breaking down ‘friction of distance’ etc (McGee 2008). The lessons from studying Taipei area can be important in understanding dynamics of land prices in other big metropolitan areas, especially when all developing countries in the world are experiencing rapid urbanization. Almost 70 percent of the world population is going to live in urban areas by 2050 according to the United Nations Expert Group Meeting (UN/POP/EGM-URB 2008).

The structure of the paper is as follows: in Section 2, related theories and literature are briefly reviewed. Section 3 lays out the empirical framework with the data described in the following section. Section 5 presents our analysis and finally, Section 6 provides some concluding remarks.

## 2 Relevant literature

Explanations for co-movements of regional house prices are manifold, complex and could be drawn from social, economic and political linkages among regional territories. Meen (1999) provides four possible explanations that may lead to spillover effects among regional housing markets – inter-regional migration, equity transfer, spatial arbitrage and local economic developments. Jones and Leishman (2006) also indicate that ripple effects are partly caused by spatial arbitrage process through household migration between local housing markets. They believe that various determinants of house prices in local housing markets (i.e. regional heterogeneity) result in a multitude of spatial patterns, and as a result, it may influence household migration.

Dieleman and Wegener (2004) also note that good accessibility can expand new residential developments into suburban areas causing more urban sprawl. It is reasonable to assume that if increases in the demand for residential land in low density areas lead to greater urban sprawl, then these areas would exhibit more prominent presence of lead-lag relations in land values. In addition, Brueckner (2000) and O’sullivan (2009) argue that rising incomes may create more demand for larger spaces, and low commuting costs boost demand for space in distant locations where land is relatively cheap. This would cause urban expansion or urban sprawl. Couch and Karecha (2006) caution that in order to control urban sprawl, any restrictions imposed on the housing supply in those peripheral areas can only increase house prices leading to less affordability. These views are largely corroborated by the empirical findings in the literature. For example, Oikarinen (2006) points out that if regional housing markets can act as substitutes for each other such as core urban and surrounding areas, an increase in house price in the centre could raise house prices in the surrounding areas with a time lag.

International evidence on regional house price dynamics is substantial. Previous studies have variously explored the possibility of ripple effects across several countries. In the UK, MacDonald and Taylor (1993), Alexander and Barrow (1994) and Ashworth and Parker (1997) provide evidence of convergence between regional house prices over the long run. Studies in the US also report significant diffusion effects among different housing submarkets (Clapp and Tirtiroglu 1994; Tirtiroǧlu and Clapp 1996; Pollakowski and Ray 1997). Stevenson (2004) uses quarterly data from 1978 to 2002 to investigate the co-movements of housing price in Dublin, Cork, Waterford, Limerick Galway and Northern Ireland. The findings reveal that due to the centrality of Dublin in the Irish economy, the ripple effect occurs from the capital to contiguous regions and then to the peripheral areas. Oikarinen (2006) also supports these findings indicating existence of substantial lead-lag relations between house price changes in the main economic centre and surrounding regions of the Helsinki metropolitan area. Luo et al. (2007), applying data from 8 capital cities in Australia, find notable ripple effects across submarkets. They suggest that the Sydney house prices only have impact on Melbourne. House prices in Adelaide and Perth not only Granger cause house price movements in Melbourne but also influence Canberra, Brisbane, Hobart and Darwin housing markets. Shi and Hargreaves (2009) also provide similar evidence that specific economic conditions at the regional levels are associated with the ripple effects, and the ripple effects are likely to spread within inter-urban centres instead of between regional centres in New Zealand. More recently, Lee and Chien (2011) employing quarterly data from 1993 to 2009 in 5 main metropolitan areas in Taiwan, indicate that except for Taipei City, house prices in other main regions exhibit causal relationships. Gray (2012) provides evidence of district level spatial spillover of house price growth. Balcilar et al. (2013) studies South African market and provides strong evidence of the ripple effects across five markets. Lean and Smyth (2013) provide an interesting case study of the ripple effects across various house types in Malaysia.

The majority of the empirical studies discussed above were based largely on causality, co-integration tests or error correction modelling (ECM) framework to estimate the interdependences of regional house prices. However, with the recent developments in spatial econometrics, it is understood that spatial patterns may not only be significant but also can alleviate several sources of estimation biases, which however, has been largely ignored and rather under-explored in this area of research. As Anselin (1999) has discussed, ignoring spatial autocorrelation or spatial dependence could cause non-constant error variance, and the results may be misleading and substantially biased when estimation strategies use standard econometric approaches to spatial issues. Few recent studies have explored the spatial dimension of the issue of ripple effect. Brady (2011) using data from 31 California counties and Holly et al. (2011) employing data for 12 UK regions suggest that the ripple effects could be examined appropriately over time and space with due consideration given to spatial dependence. Brady (2011) applies spatial panel data with spatial impulse response functions, and shows how a shock to house prices can propagate through regions over time. The analysis of Holly et al. (2011) uses spatial and temporal approaches, and they find that transitions of shocks are derived from specific regions and spatial effects. Shocks to London would spread to other regions over time and space. It suggests that the shock effects from London to other regions would last much longer if the region is further away from London. However, Brady (2011) ignores the presence of space-time covariance which may lead to violation of the stability conditions. Debarsy et al. (2012) present a more general model based on earlier works by Anselin (2001), Yu et al. (2008) and Parent and LeSage (2012) that controls for space-time covariance as well as spatially lagged exogenous variables. In this paper, we apply several such specifications to test the hypotheses.

In summary, there has been a substantial body of research in co-movements of regional housing markets, and shows causal relationships between or within regions. However, limited attention has been paid to the significance of spatial effects across regional housing markets and examination of how such effects may influence house prices. The main contribution of the current paper is to explore spatio-temporal dimension of the diffusion process using Taipei region as a case study following a dynamic spatial panel data modelling framework.

## 3 Methodology

In this study, we examine dynamic relationships in the house price movements in central and surrounding regions in Taipei. We apply several dynamic spatial-panel data methods to estimate how national and local conditions affect prices in housing sub-markets. We start with outlining the standard panel models following with a detailed discussion of the spatial models.

### 3.1 Standard panel data models

A typical bias in estimations with the panel data is the presence of unobserved heterogeneity, which may correlate with the independent variables and the residual term. For demonstration, the standard panel data model can be represented as:
1

where *y*_*it*_ indicates the house price in the *i*^*th*^ housing market in year *t. α*_*i*_ denotes the time-invariant individual effect allowing region-specific characteristics such as location, local conditions and economic structure. *x*_*kit*_ is the *k*^*th*^ factor in the *i*^*th*^ housing market in *t*, and *β* is the coefficient vector; *ε*_*it*_ is the error term. If *α* is constant, the model will be a conventional linear regression model and OLS can serve as an appropriate method to estimate the parameters. On the other hand, if correlation exists between *α* and independent variables, it can lead to the typical problem of omitted variable bias in panel data. The panel data models provide efficient tools to address unobserved heterogeneity by controlling for the fixed effects. Therefore, with potential region-specific effects across housing markets, a two-way error components model incorporating both the region-specific and the time-specific fixed effects can be employed to examine the regional housing markets. The region-specific fixed effect implies that individual factors may vary across regions but are time-invariant, and more importantly, it could have long-term effects on the housing markets. Time-specific fixed effect specification indicates that specific period of time would cause short-term disequilibrium in the housing markets but these effects may not vary across regions. Fixed effect model can be seen as a Least Square Dummy Variable (LSDV) formulation because it uses dummy variables to estimate the unobserved heterogeneity, which is tantamount to the mean-differencing approach. The model can be expressed as:
2

where *μ*_*i*_ is the region-specific constant term that is time-invariant. The model can be augmented to a two-way fixed effects model if we add the time effects *λ*_*t*_ in the equation as well:
3

Moreover, if the residuals *ε*_*it*_ exhibit temporal autocorrelation or the dependent variable *y*_*it*_ shows high persistency, dynamic panel model would work better because it allows feedbacks from current or past shocks. Therefore, equation (3) can be written as in a dynamic set-up:
4

However, the estimator is inconsistent and biased in dynamic models by using LSDV method due to existence of correlations between lagged values of independent variables and residual terms (Roodman 2009). The bias would turn out to be worse when the autoregressive coefficient is high or the number of time periods is short. Therefore, we turn to dynamic panel modelling with controls for spatial correlation.

### 3.2 Dynamic panel-spatial model

In this paper, we are interested in exploring the presence of spatial patterns or correlations. Therefore, we include a spatially lagged dependent variable to capture the spatial correlation between regions. It implies that the value of the dependent variable is jointly determined by the neighbouring units and local characteristics (Elhorst 2010). Then, the one-lagged spatial panel model can be expressed as:
5

where *ρ* is the coefficient of spatial autoregressive term and *Wy*_*jt*_ is called a spatial lag as a weighted average of observations on the variable over neighbouring units. *y*_*it*−1_ is the lag of the dependent variable, ϕ the autoregressive time dependence parameter and *w*_*ij*_ is the *N × N* spatial weight matrix. The spatial matrix *W* is pre-determined by contiguity, where the value of the spatial correlation is 1 if the region *i* and region *j* are neighbours, otherwise the value is 0. The spatial matrix is normalised with each row summing up to unity. The stability condition is: (|*ρ| + |*ϕ*| < 1*).

Due to the correlation between the spatial regressor *w*_*ij*_*y*_*jt*_ and the error term, the estimation of standard fixed effects models could be inconsistent. There are several approaches suggested in the literature with varied levels of merits and demerits (for example, see Kuethe and Pede 2011; Beenstock and Felsentein 2007 for Vector Auto-regression approaches). There are two major methods - maximum likelihood (MLE) and instrumental variables or generalised method of moments (IV/GMM) approaches - that are used to deal with the spatial interactions. However, considering the complex moment conditions in GMM and a lack of a direct GMM estimator for the spatial dynamic-panel model, an instrumental variable approach within a two-stage estimation process has also been suggested in the literature (Brady 2011).

However, the simple SAR formulation in equation (5) has limitations due to the absence of effective control for potential space-time covariance. Ignoring the space-time covariance may lead to violation of the stability condition (|*ρ| + |*ϕ*| <* 1). Therefore, based on works by Anselin (2001), Yu et al. (2008) and Parent and LeSage (2012), Debarsy et al. (2012) present a more general dynamic *spatial lag* panel model that allows for time and spatial dependence both as well as a cross-product term reflecting spatial dependence at a one-period time lag. They also add spatially lagged exogenous variables to the set of covariates, leading to a dynamic *Spatial Durbin Model* (SDM).
6

where *ρ* the spatial dependence parameter, ϕ the autoregressive time dependence parameter, and *θ* the spatio-temporal diffusion parameter. *ϵ*_*it*_ is assumed *i.i.d*. across *i* and *t* with zero mean and constant variance. The stability condition is: (|*ρ*| *+* |ϕ| *+* |*θ*| *< 1*). However, this stability condition may be too restrictive in many cases (Elhorst 2012). However, a less restrictive condition may also be applied counting the negative values.

Furthermore, the direct (or, own), indirect (or, spillover) and total effects can be estimated from spatial models by computing partial derivatives of the impact from changes to the variable. In the most general *Spatial Auto-Regressive* (SAR) model, the equation (6) could be rewritten in vector form in one housing characteristic. Thus the derivative of *Y* with respect to *x*_*k*_′ is as:
7

The marginal effect is derived as (see Elhorst 2014):
8

*β*_*k*_ is the marginal implicit price, but the marginal implicit price of the SDM is [*β*_*k*_ *I* + *γ W*](*I* − ρ *W*)^− 1^. The house price in location *i* could be affected by both of a marginal change of one housing characteristic in location *i* and marginal changes of housing in the other locations. The former is called the direct or own effect and the later an indirect or spillover effect. When both *ρ* and *γ* are equal to zero, the indirect effects do not exist. The indirect effects also known as spillover effects due to from an observation’s neighbourhood set, but the effect of *x*_*jk*_ on *y*_*j*_ is also zero if the element *w*_*ij*_ of the spatial weights matrix is zero (Elhorst 2012). According to LeSage and Pace (2009), the direct effect could be estimated by the average of the diagonal elements, and the indirect effect measured by the average of the row sums of non-diagonal elements of the matrix.

In our estimation framework, we employ several specifications: (1) Brady (2011) SAR model; (2) Debarsy et al. (2012) SDM model without time effects; (3) Debarsy et al. (2012) SDM model with time effects; and (4) Debarsy et al. (2012) SDM model with time effects to calculate direct, indirect and total effects.

## 4 Data description

**Figure 3:**
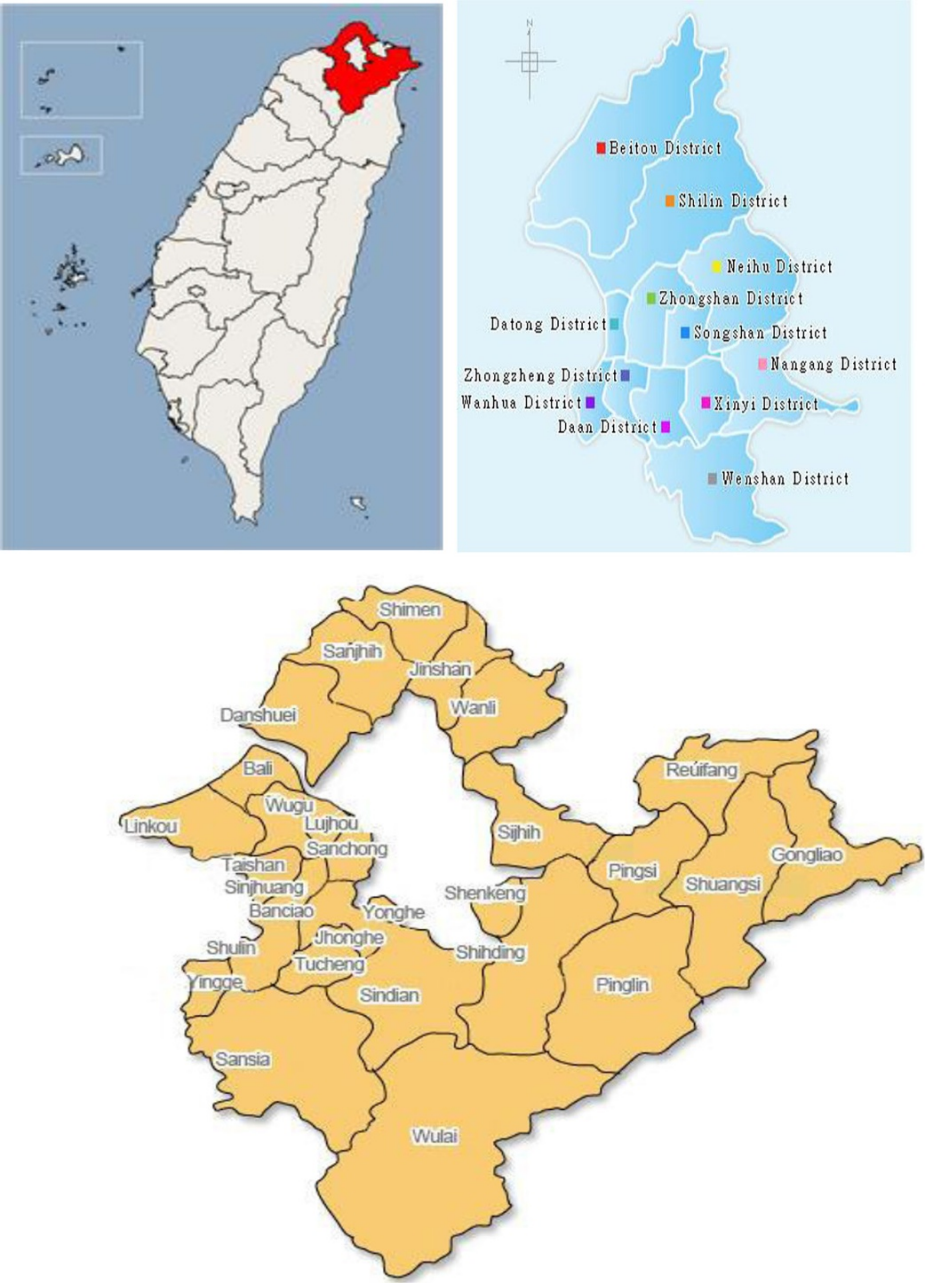
**41 Areas in Taipei.**

Figure 3 is the map of Taipei showing all 41 local areas that we analyse in this paper. We divide 41 Taipei local areas or housing sub-markets into five regions in Table 1, namely Central Taipei City (CT), the rest of Taipei City (RT), Satellite City (SC), Western Periphery (WP) and Eastern Periphery (EP) to investigate and compare the house price movements. We also combine these five regions into more coherent clusters according to administrative boundaries. Specifically, we combine CT and RT into a region-cluster; CT, RT, ST into a region-cluster; WP and EP into a region-cluster. The central Taipei city is the central geographic area in Taipei city, while the rest of Taipei city is referred to the other areas of Taipei city. Areas contiguous to Taipei city are named as Satellite city because these areas could be seen as extended areas of the Taipei city due to improved transportation and low commuting costs. The annual data is obtained for all 41 local areas in Taipei from 1992 to 2010. Therefore, in panel models, total number of observations is 738. In Central Taipei city, there are 5 local areas consisting of 90 observations; in the Rest of the Taipei city, there are 7 local areas consisting of 126 observations; in Satellite city, there are 11 local areas consisting of 198 observations; in the West Peripheral region, there are 7 local areas with 126 observations and in the East Peripheral region there are 11 local areas with 198 observations*.* Tables 2, 3 and 4 report descriptions of the variables, summary statistics and sprawl index respectively.

The trends of real residential land prices in Taipei City and New Taipei City are presented in Figures 1 and 2. It shows that the Taipei residential land prices declined gradually from 1991 to early 2000s due to weak economic performance. Land prices dropped to the lowest because of the SARS infection in 2003 and climbed steeply since 2004 with deregulation and low interest rate regime. The slowdown in 2008 resulted from the global financial crisis (GFC). However, due to a strong stock market performance, low tax and interest rate regime, and a stable political relationship between Taiwan and China, the land prices registered growth since 2009.

Furthermore, Taipei City’s housing market is more stable compared to other four regions. It is probably due to the limited housing supply and rising demand from investment community. These forces may be causing excess demand in the central region, and thus, leading to downward stickiness or rigidity in house prices. On the other hand, other growth centres in the surrounding areas showed relatively high volatility in prices possibly due to substantial substitution effects, good transportation network and population movement towards the suburbs. Excess demand for housing not only pushes the prices in Centre but may also affect surrounding regions. However, when house prices decline, investors may prefer to hold onto central region’s properties (as safer alternatives) rather than those in the surrounding regions. Therefore, the decline in housing demand in surrounding regions may cause further decreases in prices leading to volatile housing markets. Residential land prices are used as a dependent variable as there are no competent house price measures across all 41 local areas that we cover in this study. As Figures 1 and 2 show that the real changes in house and land prices are reasonably close in trends.

**Table 1:** **Regional housing markets in Taipei**

Regions	Total 41 areas
Central Taipei city (CT)	SongShan (SO), XinYi (XY), DaAn (DN), ZhongZheng (ZE), ZhongShan (ZA)
Rest of Taipei City (RT)	DaTong (DT), WanHua (WH), Neihu (NH), NanGang(NG), WenShan (WS) Beithou (BA), Shilin (SL)
Satellite City (SC)	Sanchong (SC), Banciao (BC), Jhonghe (JH), Yonghe (YH), Sinjhuang (SU), Sindian (SN), Tucheng (TU), Lujhou (LO), Sijhih (SI), Danshui (DS), Shenkeng (SK)
Western Periphery (WP)	Bali (BL), Wugu (WG), Taishan (TA), Linkou (LK), Shulin (SH), Yingge (YG), Sansia (SA)
Eastern	Wulai (WU), Shiding (SD), Pinglin (PL), Pingsi (PS),
Periphery (EP)	Shuangsi (SS), Gongliao (GL), Reuifang (RF),
	Jinshan (JS), Wanli (WL), Sanjhih (SJ), Shimen (SM)

**Table 2:** **Description of variable and data sources**

Variables	Description	Sources
**Dependent variable**		
Land price	Change in median residential land price/Natural log in median residential land price	Department of Land Administration
**Independent variables**		
Spatial regressor	A weighted average of neighbour’s land prices in natural log	
Income	Change in income per capita/Natural log in income per capita	Financial Data Centre, Ministry of Finance
Population density	Change in density/Natural log in density	National Statistics Taiwan

*Relevant variables are converted to real measures using CPI.

**Table 3:** **Summary statistics**

	41 areas		Central Taipei City		Rest of Taipei City		Satellite City		Western Periphery		Eastern Periphery	
	Mean	SD	Mean	SD	Mean	SD	Mean	SD	Mean	SD	Mean	SD
Land price growth	-0.001	0.057	0.005	0.058	-0.002	0.043	-0.003	0.052	-0.016	0.060	0.011	0.064
Spatial regressor	10.798	0.907	12.378	0.086	11.704	0.175	8.990	4.510	10.495	0.392	9.354	0.429
Income per capita growth	0.018	0.069	0.024	0.066	0.018	0.047	0.018	0.045	0.011	0.047	0.021	0.109
Population density growth	0.010	0.027	-0.005	0.034	-0.002	0.021	0.016	0.021	0.028	0.020	0.005	0.026

**Table 4:** **Variation in sprawl index across regions**

Region	1992	2010	1992–2001 changes	2001–2005 changes	2005–2010 changes	1992–2010 changes
Central Taipei City	45.52	45.94	0.09	0.16	0.17	0.42
The rest of Taipei City	55.67	57.36	1.16	0.35	0.18	1.69
Satellite City	38.32	44.30	5.26	0.34	0.38	6.08
Western Periphery	36.81	38.92	1.18	0.14	0.79	2.11
Eastern Periphery	33.00	33.15	-0.33	0.62	-0.14	0.15

Note: *Sprawl Index = (((S% − D%)/100) + 1)) × 50*; where, D% = percentage of the total population in high-density area; S% = percentage of total population in low-density.

A number of explanatory variables including demographic, economic and accessibility conditions are suggested by the previous studies. As Jud and Winkler (2002) point out, population growth, changes in income, construction costs and interest rates are significant determinants of house prices. When there are increases in income, population due to international, inter-regional and intra-regional migration, the demand for housing will rise and thus push up the prices. They also find that house price movements are significantly influenced by location specific fixed effects. In addition, many studies have argued that the neighbourhood characteristics have strong influences on house prices. These neighbourhood variables include location of the house relative to public transportation, historic district, education and crime (Boyle and Kiel, 2001). At the same time, several neighbourhood variables tend to be highly correlated which may raise the issues of multicollinearity. Moreover, some neighbourhood variables are often measured with error which can lead to potentially severe attenuation biases in coefficient estimates. Therefore, we opt for a parsimonious specification that include independent variables such as income per capita, population density, construction costs and also medical (number of medical personnel/1,000 population) in instrumental variable specification (see Table 2 for details).

All data is obtained at the local area level. However, the construction cost is the national series to capture the macro-economic influences on regional housing markets. Table 3 presents summary statistics across 41 local areas in Taipei. It suggests that the average growth rate of residential land price was negative and a high level of price volatility implying weak and volatile housing markets over the last two decades. It also reveals that the income per capita growth was relatively high in Central Taipei City. In addition, the number of medical personnel showed relatively high level in Western Periphery.

To understand the population movement better, we compute an Urban Sprawl index following Lopez and Hynes (2003) - *SI = (((S% − D%)/100) + 1)) × 50. SI* = sprawl index for metropolitan area; *D%* = percentage of the total population in high-density area; *S%* = percentage of total population in low-density area. The range of values for the sprawl index is from 0 to 100 as computed in a diffusion index formulation. If the value is at 100, it indicates highest level of sprawl. At 50, the distribution of the population is spread evenly. The sprawl index is used because it allows us to compute different levels of concentration and examine temporal and geographic changes and the effects of centralisation (Wassmer and Baass 2006). The main difference of the sprawl index from the population density is that it can assess how density is concentrated across areas. In this paper, our interest is to investigate how different types of urban pattern i.e. dense, centralized, decentralized and extremely decentralized could have an impact on land prices, which can be effectively revealed by the sprawl index.

According to Table 4, the Rest of Taipei City has a sprawl index higher than 50, suggesting the population concentration in relatively low-density areas. In contrast, the West and East peripheral areas exhibit relatively less level of sprawl and the possible explanations for this centralisation may be attributed to the geographic features, land-use policies, transportation network and local economic demand shifters. Moreover, the changes over 1992–2001 reveal high levels of sprawl in the Rest of Taipei City and Satellite city indicating greater growth in low-density areas in these regions. The high level of sprawl could have resulted from the completed transportation system with improved accessibility and thus removing the barriers for population movement and housing development.

## 5 Results and analysis

### 5.1 Granger causality

While it is not central to this paper to take time-series approach, we test for Granger Causality to identify potential spatial relationships. It must be noted, though, that these matched pair tests may significantly understate the spatial effects. The bivariate causality results are presented in Table 5 showing the vertical series Granger causing the horizontal series. According to the Granger causality, when past values of X_1_ contains information that helps predict X_2_ above and beyond the information contained in past values of X_2_ alone, a process X_1_ is said to *Granger-cause* the process X_2_ (see Granger and Newbold 1974; Granger 1988). There are some interesting findings revealed in these relationships. Firstly, although prices in central cities may have influences on some of the other regions, prices in some regions, like in new growth areas, lead to house price changes in the centres. The finding refutes the assumption that past house price movements in centres may predict current suburban housing markets. As Oikarinen (2006) argues, the substitution effects between core central regions and surroundings may drive overall direction in the market. These contiguous areas could act as extended areas of the central market due to improved transportation and low commuting costs which alleviate the severity of mobility constraints and thereby allow easier movement of key factors of production i.e. labour and capital. Moreover, the higher house prices in the centres also affect the direction of migration to suburban or peripheral areas, and it may trigger house price changes in those areas potentially leading to house price movements in the centre. Another interesting finding is that the contiguity feature appears to dominate these inter-relationships across housing markets. If two cities are contiguous or close to each other, there exist positive feedback effects. It implies that adjacent area’s lagged house prices would have noticeable impacts on the contiguous regions (Stevenson 2004; Hui, 2010). This clearly indicates the need to control for spatial dependence in the estimation framework with an explicit recognition of the spatial auto-correlation patterns through spatial weight matrix.

**Table 5:** **Granger causality between 41 local housing markets**

Pairwise Granger causality in Taipei City												
	**Central Taipei**					**Rest of Taipei**						
**→**	**XY**	**DN**	**ZE**	**ZA**	**SO**	**BT**	**SL**	**NG**	**NH**	**WS**	**DT**	**WH**
**XY**	-	0.54	0.514	1.430	0.862	3.083	2.901	0.247	1.081	0.046	1.422	0.225
**DN**	2.957^*^	-	0.271	1.196	0.496	0.746	7.111	0.461	1.296	1.319	2.388^*^	0.287
**ZE**	4.262	1.631	-	1.308	0.226	0.052	6.195^*^	1.732	0.732	1.496	6.099^*^	2.595^*^
**ZA**	0.792	0.293	2.102	-	0.643	2.132	2.045	0.163	3.210^**^	1.309	0.820	0.420
**SO**	0.019^**^	7.617^***^	1.254	0.001	-	3.109^**^	1.148	0.088	0.673	0.253	0.725	0.551
**BT**	2.602^*^	0.021	0.346	0.098	0.587	-	3.466^**^	0.111	0.736	0.647	1.730	0.777
**SL**	0.160^*^	5.134^**^	0.915	0.361	1.191	3.996^**^	-	0.132	1.001	1.291	0.546	0.796
**NG**	2.952^*^	0.561	1.608	2.551^***^	0.095	1.345	0.375	-	1.254	1.483	9.601^*^	0.951
**NH**	2.173	4.051^**^	1.491	0.662	1.614	1.072	7.766^**^	0.659	-	0.427	8.416^**^	4.819^**^
**WS**	5.761^**^	2.177	0.130	0.803	0.641	0.944	4.699^**^	0.485	0.307	-	5.985^**^	1.507
**DT**	3.766^**^	2.607	1.608	1.549	0.197	0.920	2.981	0.001	5.354^**^	4.026^**^	-	0.016
**WH**	1.855	0.339	0.782	3.215^**^	0.173	0.907	4.565^**^	0.078	0.566	0.234	0.455	-
**Pairwise Granger in the Satellite City**												
**→**	**BC**	**SC**	**YH**	**JH**	**SU**	**SN**	**TU**	**LO**	**SI**	**DS**	**SK**	
**BC**	-	1.603	2.022	0.711	0.427	2.611^*^	3.182^*^	0.576	0.521	1.197	0.183	
**SC**	0.190	-	0.576	0.831	0.864	1.632	1.376	2.462^*^	0.477	1.050	0.874	
**YH**	0.060	0.572	-	0.498	1.924	0.351	1.975	0.443	1.278	0.018	0.392	
**JH**	0.963	0.048	0.346	-	0.819	0.569	0.321	0.129	1.878	0.048	0.862	
**SU**	3.251^*^	0.801	1.134	0.057	-	1.190	4.556^**^	0.103	0.474	1.456	0.932	
**SN**	1.502	0.342	0.191	0.654	1.897	-	2.978^*^	0.606	0.609	0.696	0.802	
**TU**	1.278	0.318	0.553	2.004	0.167	0.191	-	0.696	0.154	1.578	0.900	
**LO**	1.847	0.222	0.577	0.876	1.415	0.345	1.103	-	0.354	1.384	4.181^**^	
**SI**	1.011	1.643	0.983	0.732	0.463	1.450	0.606	0.381	-	0.577	1.198	
**DS**	0.565	1.322	1.295	1.870	0.486	0.209	3.057^**^	2.204	1.336	-	0.389	
**SK**	0.962	1.470	1.631	1.116	0.616	4.417^**^	0.405	0.165	1.911	0.595	-	
**Pairwise Granger causality in the Western Periphery**												
**→**	**BL**	**WG**	**TA**	**LK**	**SH**	**YG**	**SA**					
**BL**	-	0.025	0.996	0.122	1.627	0.166	0.192					
**WG**	2.480^*^	-	6.631^***^	4.861^**^	3.281^*^	2.200	2.510^*^					
**TA**	8.55^***^	0.750	-	2.249	2.331	0.754	4.970^**^					
**LK**	9.305^***^	1.207	0.421	-	1.305	0.204	0.414					
**SH**	2.123	1.413	0.416	0.421	-	0.210	1.287					
**YG**	4.271^**^	0.561	2.669^*^	3.267^*^	2.834^*^	-	0.605					
**SA**	3.138^**^	0.088	1.652	0.706	0.061	0.856	-					
**Pairwise Granger causality in the Eastern Periphery**												
**→**	**WU**	**SD**	**PL**	**PS**	**SS**	**GL**	**RF**	**JS**	**WL**	**SJ**	**SM**	
**WU**	-	1.478	0.443	2.302	0.971	0.027	1.892	3.293^**^	0.940	0.263	0.662	
**SD**	1.222	-	0.665	0.836	2.865^*^	0.872	1.496	0.154	0.080	1.252	0.561	
**PL**	0.622	1.041	-	2.219	0.141	0.314	1.730	1.085	0.553	0.553	4.181^**^	
**PS**	1.425	1.536	0.327	-	2.635^*^	0.723	0.114	1.213	0.240	0.101	0.140	
**SS**	4.079^**^	0.669	0.706	0.246	-	0.031	1.287	0.108	0.130	1.179	0.596	
**GL**	2.668^***^	0.290	0.391	1.957	4.142^**^	-	0.354	4.056^**^	1.178	1.245	0.466	
**RF**	2.591^***^	0.678	0.040	0.169	0.133	1.525	-	3.341^**^	0.032	0.657	1.737	
**JS**	0.893	1.233	0.224	0.492	0.827	0.334	0.452	-	0.135	1.757	0.437	
**WL**	0.644	0.373	0.224	0.275	2.041	0.016	0.266	1.918	-	3.572^**^	0.413	
**SJ**	0.849	2.608^*^	1.548	2.894^*^	1.814	2.559^*^	0.643	0.083	2.019	-	0.098	
**SM**	1.304	2.002	0.388	0.358	0.024	0.969	0.141	0.133	2.688^***^	5.423^*^	-	

Note: The bivariate results are F-statistics based on 2 lags. ‘***’, ‘**’, and ‘*’ denote 1 percent, 5 percent and 10 percent significance levels.

### 5.2 Dynamics of residential prices in spatial panel model

**Figure 4:**
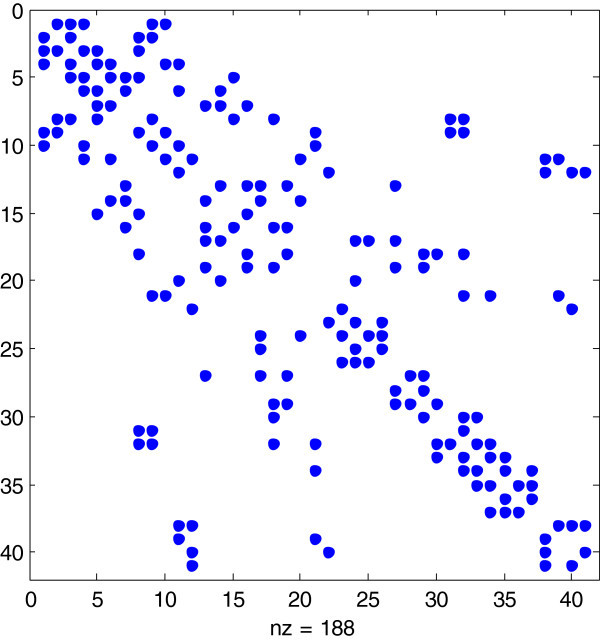
**First order spatial matrix.** Note: This figure shows the 41 × 41 first order spatial matrix for 41 areas. It represents that there exist 94 contiguous relations between areas.

Table 6 shows the results of regression including OLS and the two-stage least squares (TSLS) estimation with 41 local areas and 3 regions using a simple SAR modelling framework as represented by Equation (5). We use the spatial matrix as presented in Figure 4. All variables are expressed in natural log except for changes in construction cost. So, the coefficients can be interpreted as elasticity measures. The regression results show reasonably robust significance of the spatial regressor and the one-lagged residential land prices. For overall Taipei market encompassing all 41 local areas, a 1% increase in spatial regressor would increase residential land prices by almost 0.18%. A 1% increase in residential land prices in the previous period would also have a positive effect of around 0.78% on the current land prices. These findings confirm that, as in the previous research, spatial dependence has a strong positive impact on regional housing markets. When we look through all regions, the spatial regressor takes on positive sign with varying level of significance and size. The same holds true for the one-lagged residential land price.

**Table 6:** **Dynamic panel-spatial models: simple SAR model**

	Taipei (all areas)	Central Taipei City	Rest of Taipei City	West Peripheral region
	(1)	(2)	(3)	(4)
Spatial regressor	0.177***	0.512***	0.155**	0.285**
	(4.494)	(4.289)	( 2.640)	( 2.465)
Land Price(lag1)	0.786***	0.623***	0.864***	0.773***
	( 26.124)	(6.323)	(11.584)	( 10.236)
Adj. R^2^	0.996	0.938	0.949	0.990

Note: Robust *t* statistic is reported within the parentheses. ‘***’, ‘**’, and ‘*’ denote 1 percent, 5 percent and 10 percent significance levels. All models are estimated using two-stage IV procedure with area fixed effects and other local area controls such as income per capita, sprawl and construction cost index. For IV, the instruments include one period spatial lags of per capita income, sprawl, medical and the spatial regressor lagged one period. The sprawl is calculated as: *Sprawl Index = (((S% − D%) ⊠ 100) + 1)) × 50*. Where, D% = percentage of the total population in high-density area; S% = percentage of total population in low-density. Models (2), (3) and (4) show that the stability condition of (*ρ* + *ϕ <* 1) is violated i.e. (*ρ* + *ϕ >* 1).

However, as discussed under the Methodology section, a key concern with Equation (5) (and, results of Table 6 is the possibility of violation of the stability condition. Indeed, in models (2), (3) and (4) in Table 6, sum of the coefficients is greater than 1, thus violating the key stability condition (|*ρ|* + |*ϕ| <* 1). This is due to a lack of control for the space-time covariance in Equation (5). Therefore, we next move onto more general *Spatial Durbin Model* (SDM) as represented by Equation (6).

Table 7 reports SDM models of All Taipei region (including all 41 local areas) and also the results for three combined region-clusters. We first show the model without time fixed effects for All Taipei in column (1). However, Taipei has experienced several government interventions over last couple of decades that could affect the housing markets. Therefore, the time fixed effects should be included to control for any such shifts in the intercept. Column (2) reports the model for All Taipei with time dummies. As expected, the results are different and reveal importance of the inclusion of the time fixed effects. Specifically, without controlling for the time fixed effect, a 1% increase in neighbouring region’s land price will lead to 0.28% increase in All Taipei. However, the effect size reduces to about 0.19% when we control for the time fixed effects in column (2). The neighbouring local area’s space-time regressor becomes insignificant in column (2). However, effect of own lagged land price remains quite robust with time fixed effects. The same holds true for income and population density. Specifically, a 1% increase in the previous period’s land price will have almost 0.8% increase in the current period’s land price. We also find that a 1% increase in income and population density will lead to statistically significant effect of almost 0.14% and 0.09% increases in land prices respectively. However, the neighbouring local area’s income and population density are not significant in column (2). Moreover, across two models in Table 7, we see that the most restricted stability condition (i.e. |*ρ| + |ϕ| + |θ| < 1*) for Equation (6) is satisfied. In fact, when we control for the time fixed effects in model 2 for all local areas, the stability condition is almost satisfied in its most restrictive form. Overall, we do find that the inclusion of time effects in the model is important.

**Table 7:** **Dynamic Spatial Durbin Models**

	All Taipei (41 areas)	All Taipei (41 areas)	Core Taipei City	Core Taipei City including Satellite City	East and West Peripheral regions
	(1)	(2)	(3)	(4)	(5)
Spatial regressor W*Land Price	0.285***	0.188***	-0.471***	0.021	0.133**
	(5.948)	(3.728)	(-5.268)	(0.301)	(2.247)
Space-time regressor: W*Land Price(lag1)	-0.178***	-0.066	0.384***	-0.068	-0.035
	(-3.314)	(-1.155)	(3.791)	(-0.839)	(-0.503)
Land Price(lag1)	0.795***	0.800***	0.703***	0.735***	0.827***
	(37.831)	(38.706)	(15.023)	(23.344)	(26.769)
Per Capita Income	0.125**	0.140**	0.236**	0.032	0.208**
	(2.248)	(2.63)	(2.063)	(0.365)	(2.489)
Population Density	0.084	0.091*	0.079	0.069	0.057
	(1.576)	(1.747)	(1.098)	(0.933)	(0.631)
W*Per Capita Income	-0.123**	0.114	-0.182	-0.302	0.052
	(-1.964)	(1.125)	(-0.602)	(-1.638)	(0.441)
W*Population Density	-0.159**	-0.113	0.047	-0.241	-0.058
	(-2.131)	(-1.334)	(0.311)	(-1.413)	(-0.483)
Model Description	Spatial Fixed Effects	Spatial & Time Fixed Effects	Spatial & Time Fixed Effects	Spatial & Time Fixed Effects	Spatial & Time Fixed Effects
N	738	738	216	414	324
σ^2^	0.0056	0.0054	0.0017	0.0038	0.0071

Note: All variables are specified in log. *t* statistic is reported within the parentheses. ‘***’, ‘**’, and ‘*’ denote 1 percent, 5 percent and 10 percent significance levels.

It is quite likely that a mega-urban metropolitan area such as Taipei contains several geographic clusters with distinct economic and spatial dynamics. Therefore, we also look at three combined region-clusters to check robustness of the full sample results. Specifically, first we combine the local areas in the main Taipei city and then also add the Satellite City region. We also combine the peripheral regions of East and West, which constitutes the New Taipei City. We have also run models with individual regions but for the brevity of reporting, we only present these three combinations of region-clusters. Quite remarkably, the results do reveal interesting departures from the All Taipei results of column (2). Most notably, the spatial regressor is significant but negative in the Core Taipei City (column (3)). A 1% increase in neighbouring local area’s land price will lead to a fall of almost 0.47% in a local area’s land price in the Core Taipei City. Although apparently puzzling, a couple of explanation can be provided for such effect. While the negative result of the spatial regressor is not common, but it is not unprecedented either in the literature (Holly et al. 2011). A plausible explanation is the uniqueness of the region in the question i.e. the Core Taipei City is very unique with its urban-economic characteristics as a capital city and own government structure. Moreover, it may probably be due to the prevalence of speculative activities in the core city area. A high future price expectation may drive up the housing demand in the area with resulting lack of demand in neighbouring local areas. This reasoning is supported by positive and significant space-time covariance. However, when we expand the geographic expanse by adding the Satellite City, the spatial dependence is no longer significant. This is probably caused by a dominant substitution effect due to modernised transportation system in mid-1990. The result for the Peripheral region shows expected and significant positive effect of spatial regressor. Specifically, a 1% increase in neighbouring local area’s land price will cause almost 0.13% increase in an area’s land price. This small but statistically significant positive feedback is expected in the peripheral regions due to emerging opportunities and population growth.

The most robust result in Table 7 is for the previous period’s land price. This temporal effect is almost 0.7–0.8% for a 1% increase in previous period’s land price. Income and population density show positive feedback effects, albeit with varying degree of significance. This finding conforms to other findings in the literature (e.g. Kahn 2001). A plausible reason is that the Asian newly-industrialising countries (NICs) have been quite successful in breaking down the ‘friction of distance’ and thus reducing the commuting cost (McGee 2008). Taipei local areas are well connected by modern transportation system. Therefore, suburban areas have become more desirable to households and thus raising the demand for housing. This lends support to proponents of ‘Smart Growth’ policies. The same is true for the predominantly manufacturing areas of Western Peripheral region and predominantly farmland areas of Eastern Peripheral region. It should be noted though that these findings are by no means definitive and are rather strongly indicative of the complexities that surround this area of research (see arguments in Mills 2002). We did not find much effect for the neighbouring local area’s income and population density from models (2) to (5). While we have presented a number of modelling frameworks in this paper, this is, by no means, a complete list of plausible models in this area. Several other approaches have been adopted and suggested in the literature. We provide a number of the model to show a general pattern in the results.

Next we look into the detailed break-down of effects of income and population density in terms of direct (or, own), indirect (or, spillover) and total effects. Specifically, we compute these effects according to Equations (7) and (8). Table 8 reports the direct, indirect and total effects of the coefficient estimates from Table 7 columns (2) to (5). Direct effects computed in Table 8 come close to the standard coefficients in Table 7. The results do not reveal much significance in terms of indirect or spillover effects. Although the spillover effect is not statistically significant, those have affected the total effect in a substantial way across many regions. For example, the All Taipei result reveals a direct effect of 0.15% increase due to 1% increase in income. We also find that the income is associated with an indirect or spillover effect of almost 0.16% albeit statistically insignificant. However, the upshot is much stronger and bigger total effect of almost 0.32%. In the same vein, the population density total effect is heavily influenced by opposite signs of direct and indirect effects in Taipei. The choice of suburban or sparsely populated locations is driven by socio-demographic or spatial factors (Ravenstein 1885; Skeldon 1997). These factors include relatively higher demand for labour, better economic opportunities and availability of land encouraging migration. Moreover, the different cost of living between regions leads to migration and the growth in resident population pushes housing values upwards. However, the relatively high level of house prices also thwarts further migration because of increasing housing cost that dents affordability (Potepan 1994; Cameron and Muellbauer 1998; Jeanty et al. 2010). Taipei City, being the dominant region of the national economy, experienced a large population growth of around 42%. However, since 1991 the negative population growth in Taipei City and rapidly growing population in satellite cities or the western periphery suggest population movement to surrounding areas or suburbs possibly triggered by higher housing cost. Overall, Table 8 reveals that income and population density are not the primary demand shifters of the housing markets in most of Taipei regions. As Cameron and Muellbauer (1998) suggest, the determinants of contiguous region migration could be strongly decided by relative housing market variables when cheap commuting is another choice. The Taipei metropolitan area has undergone significant developments in terms of public transportation system, which may have weakened the standard income and population effects on house prices. However, the spatial dependence is significant across all areas, which may indicate strong substitutability across the Taipei local areas. The existence of spatial effects among Taipei local areas conforms to our theoretical expectation.

**Table 8:** **Dynamic Spatial Durbin Models – effects computation**

	All Taipei (41 areas)	Core Taipei City	Taipei city including Satellite City	East and West Peripheral regions
*Direct effect:* Per capita income	0.155***	0.267**	0.027	0.213**
	(2.872)	(2.233)	(0.292)	(2.579)
*Indirect effect:* Per Capita Income	0.166	-0.219	-0.315	0.086
	(1.516)	(-0.91)	(-1.608)	(0.671)
*Total effect:* Per Capita Income	0.321***	0.048	-0.288	0.299**
	(2.986)	(0.208)	(-1.296)	(2.48)
*Direct effect:* Population Density	0.088*	0.077	0.067	0.053
	(1.766)	(0.901)	(0.87)	(0.615)
*Indirect effect:* Population Density	-0.116	0.008	-0.246	-0.055
	(-1.289)	(0.056)	(-1.379)	(-0.422)
*Total effect:* Population Density	-0.028	0.085	-0.179	-0.002
	(-0.351)	(0.945)	(-1.252)	(-0.014)

Note: All variables are specified in log. *t* statistic is reported within the parentheses. ‘***’, ‘**’, and ‘*’ denote 1 percent, 5 percent and 10 percent significance levels. These effects correspond to the parameter estimates in Table 7, columns 2, 3, 4 and 5.

## 6 Conclusion

This study investigates dynamics of the residential land prices across 41 local areas in Taipei by using panel data frameworks and dynamic spatial panel model over the period of 1992–2010. The Granger Causality tests show that the prices in the Central regions have impacts on the prices of the surrounding areas. However, some regions, particularly the new growth local areas, lead house price increases in the city centres possibly due to strong substitution effects. These new growth cities can be viewed as substitutes for housing in the centre because of the cheaper housing options and easy accessibility to the centres due to improved transportation system in Taipei. Furthermore, contiguity plays an important role in inter-relations of house prices implying the existence of strong feedback effect between contiguous regions.

We apply several dynamic spatial panel models. The most general formulation of the spatial autocorrelation model suggests that the spatial dependence has strong positive impacts in Taipei housing markets. This can be partly explained by the fact that an improved transportation system removes mobility constraints, opens up land for housing developments, and increases the accessibility across regions and therefore, this may lead to significant spatial effects. In spatial panel data models, the empirical results suggest that specific area and time effects could have significant influences on local housing markets. It also reveals that lagged residential land price changes have positive correlations across all areas.

The findings provide us with important implications for the policy making process in terms of urban spatial planning. The positive association of population density and land prices raises an interesting question regarding support for government interventions to impede the sprawling of urban areas. While the standard arguments regarding costs associated with sprawl (e.g. in terms of greater traffic congestion, segregation, air pollution and loss of open space) are not explicitly tested in this paper, nonetheless, it shows that a good transportation system offering reduction in commuting costs may substantially offset the negative impacts on prices due to increasing suburbanization.

We find significant support for the existence of diffusion effect in Taipei metropolitan area which implies a local area’s land price movement could be predicted not only by its own previous prices but also by other neighbouring local area’s price movements. Moreover, in the most general framework, we also find neighbouring local area’s local attributes to have some significant explanatory power. This study suggests that the local area housing market dynamics, interaction of neighbouring areas and spatial patterns should be considered when decisions regarding housing policies are made and policies are implemented, instead of focusing solely on the local housing market situation in isolation. These results may especially be more pertinent in dense, mega-urban regions undergoing rapid urbanisation, significant infrastructure developments and thus raising connectivity and spatial substitutability, which are quite common phenomena across the developing economies.

## References

[CR1] Alexander C, Barrow M (1994). Seasonality and cointegration of regional house prices in the UK. Urban Stud.

[CR2] Anselin L (1999). The future of spatial analysis in the social sciences. Geogr Inform Sci.

[CR3] Anselin L, Baltagi BH (2001). Spatial econometrics. A companion to theoretical econometrics.

[CR4] Ashworth J, Parker SC (1997). Modelling regional house prices in the UK. Scot J Polit Econ.

[CR5] Balcilar M, Beyene A, Gupta R, Seleteng M (2013). ‘Ripple’ effects in South African house prices. Urban Stud.

[CR6] Beenstock M, Felsentein D (2007). Spatial vector autoregressions. Spatial Econ Analysis.

[CR7] Bertaud A, Malpezzi S (2003). The spatial distribution of population in 48 world cities: Implications for economies in transition.

[CR8] Boyle MA, Kiel KA (2001). A survey of house price hedonic studies of the impact of environmental externalities. J Real Estate Lite.

[CR9] Brady RR (2011). Measuring the diffusion of housing prices across space and over time. J Appl Econom.

[CR10] Brueckner JK (2000). Urban sprawl: diagnosis and remedies. Int Reg Sci Rev.

[CR11] Cameron G, Muellbauer J (1998). The housing market and regional commuting and migration choices. Scot J Polit Econ.

[CR12] Can A (1990). The measurement of neighborhood dynamics in urban house prices. Econ Geogr.

[CR13] Canarella G, Miller S, Pollard S (2012). Unit roots and structural change: an application to US house price indices. Urban Stud.

[CR14] Clapp JM, Tirtiroglu D (1994). Positive feedback trading and diffusion of asset price changes: evidence from housing transactions. J of Econ Behavior Org.

[CR15] Couch C, Karecha J (2006). Controlling urban sprawl: some experiences from Liverpool. Cities.

[CR16] Debarsy N, Ertur C, LeSage JP (2012). Interpreting dynamic space-time panel data models. Stat Methodol.

[CR17] Dieleman F, Wegener M (2004). Compact city and urban sprawl. Built Environ.

[CR18] Elhorst JP (2010). Spatial panel data model.

[CR19] Elhorst JP (2012). Dynamic spatial panels: models, methods, and inferences. J Geogr Sys.

[CR20] Elhorst JP (2014). Spatial econometrics: from cross-sectional data to spatial panels.

[CR21] Granger CWJ (1988). Some recent developments in the concept of causality. J Econom.

[CR22] Granger CWJ, Newbold P (1974). Spurious regressions in econometrics. J Econom.

[CR23] Gray D (2012). District house price movements in England and Wales 1997–2007: an exploratory spatial data analysis approach. Urban Stud.

[CR24] Holly S, Pesaran HM, Yamagata T (2011). The spatial and temporal diffusion of house prices in the UK. J Urban Econ.

[CR25] Hui HC (2010). House price diffusions across three urban areas in Malaysia. International J Hous Markets and Anal.

[CR26] Ioannides YM, Zabel JE (2003). Neighbourhood effects and housing demand. J Appl Econom.

[CR27] Jeanty PW, Partridge M, Irwin E (2010). Estimation of a spatial simultaneous equation model of population migration and housing price dynamics. Reg Sci Urban Econ.

[CR28] Jones C, Leishman C (2006). Spatial dynamics of the housing markets: an interurban perspective. Urban Stud.

[CR29] Jud GD, Winkler DT (2002). The dynamics of metropolitan housing prices. J Real Estate Res.

[CR30] Kahn M (2001). Does sprawl reduce the black/white housing consumption gap?. Hous Pol Debate.

[CR31] Kuethe TH, Pede VO (2011). Regional housing price cycles: a spatio-temporal analysis using U.S. state level data. Reg Stud.

[CR32] Lean HH, Smyth R (2013). Regional house prices and the ripple effect in Malaysia. Urban Stud.

[CR33] Lee CC, Chien MS (2011). Empirical modelling of regional house prices and the ripple effect. Urban Stud.

[CR34] LeSage JP, Pace RK (2009). Introduction to spatial econometrics.

[CR35] Lopez R, Hynes HP (2003). Sprawl in the 1990s: measurement, distribution, and trends. Urban Affairs Rev.

[CR36] Luo ZQ, Liu C, Picken D (2007). Housing price diffusion pattern of Australia’s state capital cities. Int J Strat Prop Manag.

[CR37] MacDonald R, Taylor MP (1993). Regional house prices in Britain. Scot J Polit Econ.

[CR38] McGee TG, Ginsburg NJ, Koppel B, McGee TG (1991). The emergence of Desakota regions in Asia: expanding a hypothesis. The extended metropolis: settlement transition in Asia.

[CR39] McGee TG (2008). Managing the rural–urban transformation in East Asia in the 21st century. Sustainability Sci.

[CR40] Meen G (1999). Regional house prices and the ripple effect: a new interpretation. Hous Stud.

[CR41] Mills ES (2002). Truly smart growth. Cornell Real Estate Rev.

[CR42] O’Sullivan A (2009). Urban economics.

[CR43] Oikarinen E (2006). The diffusion of housing price movements from center to surrounding areas. J Hous Res.

[CR44] Parent O, LeSage JP (2012). Spatial dynamic panel data models with random effects. Reg Sci Urban Econ.

[CR45] Pollakowski HO, Ray TS (1997). Housing price diffusion patterns at different aggregation levels: a comparison of alternative examination of housing market efficiency. J Hous Res.

[CR46] Potepan MJ (1994). Intermetropolitan migration and housing prices: simultaneously determined?. J Hous Econ.

[CR47] Ravenstein EG (1885). The laws of migration. J Stat Soc London.

[CR48] Roodman D (2009). How to do xtabond2: An introduction to difference and system GMM in Stata. Stata J.

[CR49] Shi S, Young M, Hargreaves B (2009). The ripple effect of local house price movements in New Zealand. J Prop Res.

[CR50] Skeldon R (1997). Migration and development: a global perspective.

[CR51] Stevenson S (2004). House price diffusion and inter-regional and cross-border house price dynamics. J Prop Res.

[CR52] Tirtiroǧlu D, Clapp JM (1996). Spatial barriers and information processing in housing markets: an empirical investigation of the effects of the Connecticut River on housing returns. J Reg Sci.

[CR53] (2008). United Nations Expert Group Meeting on Population Distribution, Urbanization, Internal Migration And Development, Population Division, Department of Econ and Social Affairs, United Nations Secretariat, New York, 21–23 January.

[CR54] Wassmer R, Baass M (2006). Does a more centralized urban form raise housing prices. J Pol Anal Manag.

[CR55] Yu J, de Jong R, Lee LF (2008). Quasi-maximum likelihood estimators for spatial dynamic panel data with fixed effects when both n and T are large. J Econom.

